# Development of Bionanocomposites Based on Poly(3-Hydroxybutyrate-co-3-Hydroxyvalerate)/PolylActide Blends Reinforced with Cloisite 30B

**DOI:** 10.3390/jfb11030064

**Published:** 2020-09-16

**Authors:** Clément Lacoste, Benjamin Gallard, José-Marie Lopez-Cuesta, Ozlem Ipek Kalaoglu-Altan, Karen De Clerck

**Affiliations:** 1 Polymers Composites and Hybrids (PCH), IMT Mines Ales, 6 avenue de Clavières, 30319 Ales CEDEX, France; benjamin.gallard@mines-ales.fr (B.G.); jose-marie.lopez-cuesta@mines-ales.fr (J.-M.L.-C.); 2 Centre for Textile Science and Engineering, Department of Materials, Textiles and Chemical Engineering, Faculty of Engineering and Architecture, Ghent University (UGent), Tech Lane Science Park 70A, 9052 Ghent, Belgium; ozlemkalaoglu@gmail.com (O.I.K.-A.); karen.declerck@ugent.be (K.D.C.)

**Keywords:** biopolymer, blends, PLA, PHBV, nanocomposite

## Abstract

In the present study, poly(3-hydroxybuturate-co-3-hydroxyvalerate) (PHBV) and plasticized polylactide acid (PLA) blends were processed by melt extrusion with different weight ratio (up to 20 wt.% of PHBV). Bionanocomposites were obtained through the incorporation of an organomodified montmorillonite (C30B) at 3 wt.%. The main features of the processing and physico-chemical characterization of films and injected samples were assessed and the influence of the components on the chemical, thermal and mechanical properties of the bionanocomposites was investigated. The results indicated that plasticized PLA/PHBV/C30B bionanocomposites present optimal mechanical properties for sanitary applications. Moreover, plasticized PLA/PHBV could lead to finely tuned biomaterials able to form electrospun nanofibers.

## 1. Introduction

Nowadays, due to environmental concerns, the substitution of non-renewable polymers by bio-based and biodegradable polymers has attracted considerable attention, especially for short term applications. In medical applications such as wound healing, the biocompatible character of the polymer is also required. In this context, biopolyesters seem to be the most promising materials gathering the ‘3B’ criteria: bio-based, biodegradable, and biocompatible. 

Among them, poly(lactic acid) (PLA) is an aliphatic linear thermoplastic derived from the fermentation of starch from renewable crops (corn, potato) or from cellulose that can be degraded by micro-organisms within few weeks under environmental conditions. PLA exhibits high transparency, thermal plasticity and good mechanical properties, making it currently the biopolymer most used for short-term applications such as packaging (films, rigid cups, cutlery, etc), sutures and medical devices. 

Poly(3-hydroxybutyrate-co-3-hydroxyvalerate) (PHBV) is also an aliphatic biopolyester synthesized by bacterial fermentation from renewable resources like sugar and vegetable oil or other microorganisms [1]. Due to its non-toxicity and biocompatible character, PHBV has been extensively used in pharmaceutical (encapsulation, drug delivery), biomedical (sutures, implant, surgical materials), packaging (containers, films) and coating applications [2]. Moreover, compared to other biodegradable polymers, PHBV shows low thermal resistance ability due to its high crystallinity.

In the recent past, market demand of those two promising candidates has seriously increased. Nevertheless, none of these two polymers can fulfill the requirement of almost all structural materials when used alone. Indeed, PLA and PHBV exhibit brittleness and efforts have been made recently to improve their ductility. Different methods can be used to increase the mechanical properties of biopolymers and to extend their range of industrial applications like random and block copolymerization, chemical modification or filler addition [3]. Among them, the blending of polymers is a simple and cost-effective approach to prepare composites of different morphologies and physical characteristics. Despite the immiscibility of PLA and PHBV [4,5,6], the physical and mechanical properties of their blends can be tailored by varying the composition. Ferreira et al. [4] have reported that adding PHBV to PLLA (poly-L-lactic acid) led to tougher materials compared to the neat samples, and the blend films showed yield behavior. Zembouai et al. [3,5] have studied the thermo-mechanical properties of PLA/PHBV blends of several ratios prepared by melt compounding and observed that the tensile stress and elongation at break decreased with increasing PHBV content, whereas the modulus increased. The less brittle blend was obtained for PLA/PHBV 75/25 with an elongation of 7% (±1.3) due to the fine spherulitic structure operated by the distribution of PHBV particles into the PLA matrix. It is then necessary to finely control the morphology of the blend to extend their properties. 

Moreover, the combined association with nanoclays is an effective strategy to improve polymer mechanical strength since the addition of nanofillers is able to widely extend the final properties of the reinforced material compared to the pristine polymers. Among them, the addition of organomodified layered silicates (OMLS) such as montmorillonites has given rise to an increasing interest [7,8,9]. As an example of reinforcement using montmorillonite, Paul et al. [7] attested the intercalation of the clays in PLA matrix, and noticed that the intercalation could also be supported by interlayer migration phenomenon for plasticized PLA matrix. Ozdemir at al. [8] also observed the good dispersion of montmorillonite (3 wt.%) into PLA/polyethylene glycol, enhancing the rigidity and the tensile properties of the nanocomposites. The preparation of OMLS-based nanocomposites with enhanced mechanical properties could lead to numerous competitive biomaterials like electrospun fibers for wound dressing. 

Although biopolyester polymers can be considerably improved with the above mentioned strategies, difficulties in improving films’ production through cost-effective processes still exist. In this sense, the electrospinning process has gained considerable attention for the production of effective biopolymer films. Electrospinning is indeed a simple, viable, and attractive method that can be used for industrial applications as it can be effectively up-scaled [10]. Concerning biomedical materials, studies have reported that electrospun nanofibers are the most effective wound dressing materials compared to sponges, hydrocolloids, and hydrogel materials. The nanoporous structure enables liquid evaporation and has excellent oxygen permeability, preventing bacteria contamination [11]. 

Therefore, the aim of this study was to develop biopolyester nanocomposites for the production of electrospun nanofibers with appropriate strength/stiffness balance. Plasticized PLA was used as matrix, whereas PHBV was used as minor phase, and organomodified montmorillonite nanofillers were used to control the mechanical strength. The miscibility, crystallization and melting behavior, as well as the mechanical properties of the bionanocomposite blends and films, were investigated. 

## 2. Materials and Methods 

### 2.1. Materials

Plasticized PLA was supplied by NatureWorks in pellet form under the trade name NP SF 141, extrusion grade. This polymer is a semicrystalline one having the following properties according to the supplier: density = 1.22 g.cm^−3^ and T_m_ = 150–160 °C, clarity = transparent.

PHBV was supplied by NaturePlast in pellet form under the trade name PHI002, injection molding grade. The average molecular weight is Mw = 203,000 g.mol^−1^ and the polydispersity index is PDI = 3.2. According to the supplier, the main properties of this polyhydroxy alkanoate are the following: density = 1.23 g.cm^−3^ and T_m_ = 170–176 °C, clarity = opaque.

Cloisite 30B (C30B) was supplied by Southern Clay Products (Gonzales, TX, USA). This powder (d_001_ = 1.9 nm) is a montmorillonite modified with bis-(2-hydroxyethyl) methyl tallow alkyl ammonium cations. C30B was dried under vacuum at 60 °C overnight before use.

### 2.2. Preparation of the Blends, Films and Nanofibers

The polymers were pre-dried at 60 °C at 24 h before molding in order to avoid hydrolytic degradation of the polymers in the extruder. The formulations of the samples are reported in Table 1. As reported in previous works [3,7,8], incorporation of 3% of montmorillonite increased the mechanical and thermal stability of plasticized PLA and was selected in this work.

**Figure 1:**
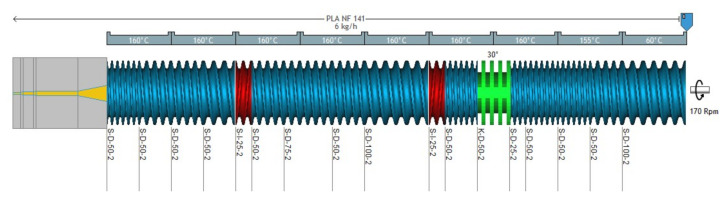
Screw profile developed from LUDOVIC® software (2016, Sciences Computers Consultants, Saint-Etienne, France).

Firstly, the biopolymer blends were prepared by melt mixing in a twin screw extruder BC21 from CLEXTRAL (CLEXTRAL SAS, Firminy, France), with the screw profile reported in Figure 1. The diameter of the screws was 25 mm and the length of the extruder was 900 mm. The blend was extruded into a single hole die of 5 mm diameter.

Beforehand the processing the polymers were dried by PIOVAN dryer at 80 °C for 12 h and in an oven at 100 °C for 12 h for C30B. The main processing parameters were fixed for all formulations as follows: the temperatures were fixed at 60 °C in the first zone (Z1), 155 °C in the second zone (Z2), and finally 160 °C from Z3 to Z12. The screw speed was fixed at 170 rpm, the flow rate was 6 kg/h, the torque was around 65 N.m-1 and the pressure was 30 bars.

Secondly, the films were prepared by a single screw extruder from Thermofisher Electron: Polylab OS7 with a Rheomex 19/25. The screw profile was a standard one 3:1 L = 25D (Diameter = 19 mm). The width of the flat die was 100 mm and the thickness was adjustable. The temperatures were Z1 = 170 °C, Z2 = 170 °C, Z3 = 165 °C, Z4 = 160 °C. The screw speed was 120 rpm. The temperature of the roll was 15 °C and the speed was adjusted manually depending of the thickness of the film. The film thickness measured by a Palmer average was 180µm along the width.

Electrospinning solutions were prepared by dissolving PLA/5P, PLA/10P and PLA/10P/3C in chloroform/DMF (*N*,*N*-Dimethylformamide) (5/1, *v/v*) solvent mixture with a concentration of 8 wt.%. Mass concentrations are expressed by weight percentages (wt.%) defined by the ratio of the polymer mass and the sum of the polymer and solvent mass. All electrospinning experiments were performed on a mononozzle setup using the solvent electrospinning technique with an 18 gauge Terumo mixing needle without bevel. A stable Taylor cone was obtained with a flow rate of 1 mL/h, a tip to collector distance of 20 cm and an applied voltage of 17 kV at room conditions ((25 ± 5) °C and (35 ± 5)% relative humidity).

### 2.3. Spectral and Thermal Characterizations

The thermal properties of the products were studied through differential scanning calorimetry (DSC), thermogravimetric analysis (TGA), and Fourier Transform infrared (FTIR) analysis. Each measurement was done in duplicate.

DSC measurements were performed on a Perkin Elmer Diamond (Perkin Elmer SAS, Villebon sur Yvette, Evry, France) operating under a constant flow of nitrogen (30 mL·min^−1^). Samples were weighed (circa 10 mg) into aluminum crucibles. Analysis in comparison to a blank crucible was performed using a temperature profile (ramp rate of 10 °C·min^−1^) from 15 °C to 200 °C. Thermal transitions were calculated from Perkin software through the tangent method.

Measurements of mass loss versus temperature were performed using a Perkin Elmer thermogravimetric analyzer (TGA) module Pyris 1 (Perkin Elmer SAS, Villebon sur Yvette, Evry, France), under N_2_ purge (flow rate of 50 mL·min^−1^). Typically, 8–10 mg of sample were placed on an aluminum oxide pan, and heated from 30 °C to 900 °C at 10 °C·min^−1^.

FT-IR spectra of PHBV/PLA/C30B blends were recorded with a Bruker Vertex 70 spectrometer (Bruker France SAS, Champs sur Marne, France) in transmission mode using a resolution of 4 cm^−1^ and 32 scans per sample. The spectra were recorded in the range of 4000–400 cm^−1^. 

### 2.4. Contact Angle

The wettability of the blends was determined using a Krüss Wettability Meter (KRUSS GmbH France, Villebon sur Yvette, France) equipped with a 60 picture/s camera. First, a 2 µL drop of water was put at the sample’s surface. Then, contact angle was determined as the mean of 10 values measured each second after drop deposition. 

### 2.5. Tensile Tests

The static tensile tests were performed at room temperature according to ISO 527 on a ZWICK TH010 universal testing machine (ZWICKROELL, Ars-Laquenexy, France) equipped with a 10 kN load head and using a loading speed of 1 mm/min. An extensometer was used at low elongation to measure the elastic moduli. The dumbbell-shape samples (75 × 4 × 1 mm^3^) were tested at least 5 times for each sample.

## 3. Results

### 3.1. Thermal Degradation 

**Figure 2:**
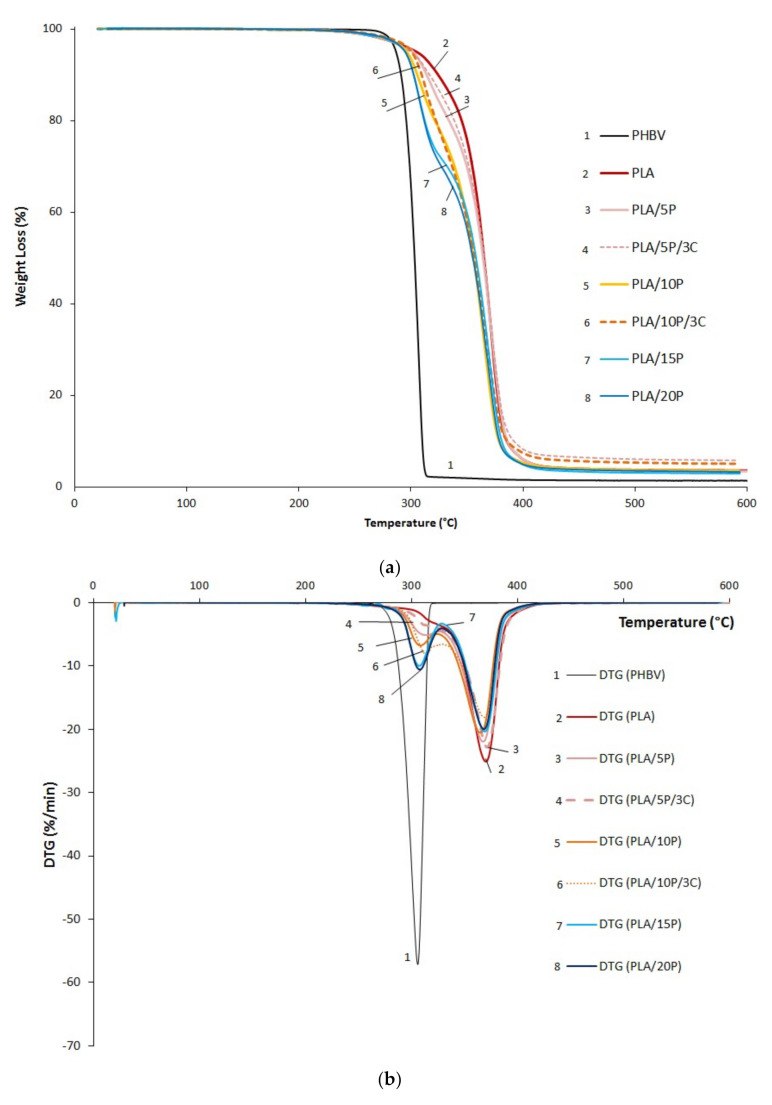
(**a**) Weight loss curves and (**b**) Derivative thermogram (DTG) of PHBV, PLA, and their blends from TGA.

The thermal stability of the pristine polymers and their blends was determined by TGA and the curves are presented in Figure 2. The decomposition temperatures at different stages (5%, 10% and 50% of mass loss) as well as residues at 600 °C are reported in Table 2. 

As expected, PLA (T_50%_ = 365 °C) is more thermally stable than PHBV (T_50%_ = 306 °C). It can be observed that the degradation of PHBV occurred in a single step separately from PLA, which presented a double peak (first peak circa 320 °C) attributed to the presence of plasticizer (Figure 2b). It was previously reported in the literature that the T_50%_ of pristine PLA is around 360 °C [3,8,12,13], which is in accordance with the measured value T_50%_ = 365 °C. However, it could be noticed that for this plasticized grade of PLA, the onset temperature T_5%_ was shifted to 301 °C compared to 330 °C reported in the literature for non-plasticized grades [3]. All the decomposition temperatures of the blends are between those of the PLA and PHBV and occurred in a two-step process. The addition of PHBV in the PLA matrix shifted the onset degradation temperature to lower values (Table 1). The presence of 3 wt.% of cloisite 30B did not significantly modify the thermal stability of the blends as similar degradation temperatures at 5%, 10% and 50% were reported for PLA/5P and PLA/5P/3C, as well as for PLA/10P and PLA/10P/3C. A very similar thermal behavior has been reported in previous studies focused on EVA-based (Ethylene-vinyl acetate) [14] and plasticized PLA-based nanocomposites [7] with an optimal thermal stability noticed at 3 wt.% of montmorillonite content. Meanwhile, the residue was increased with the presence of C30B at 5.0% for PLA/10P/3C against 3.5% for PLA/10P/3C, and at 5.8% for PLA/5P/3C against 3.4% for PLA/5P/3C. This is in agreement to the organic fraction of C30B (around 35 wt.%). Note that this grade of PLA has much more residue (3.6%) than PHBV (1.3%), suggesting the presence of additives able to produce a charred material. 

### 3.2. Thermal and Crystallization Behavior of PLA/PHBV Blends

**Figure 3:**
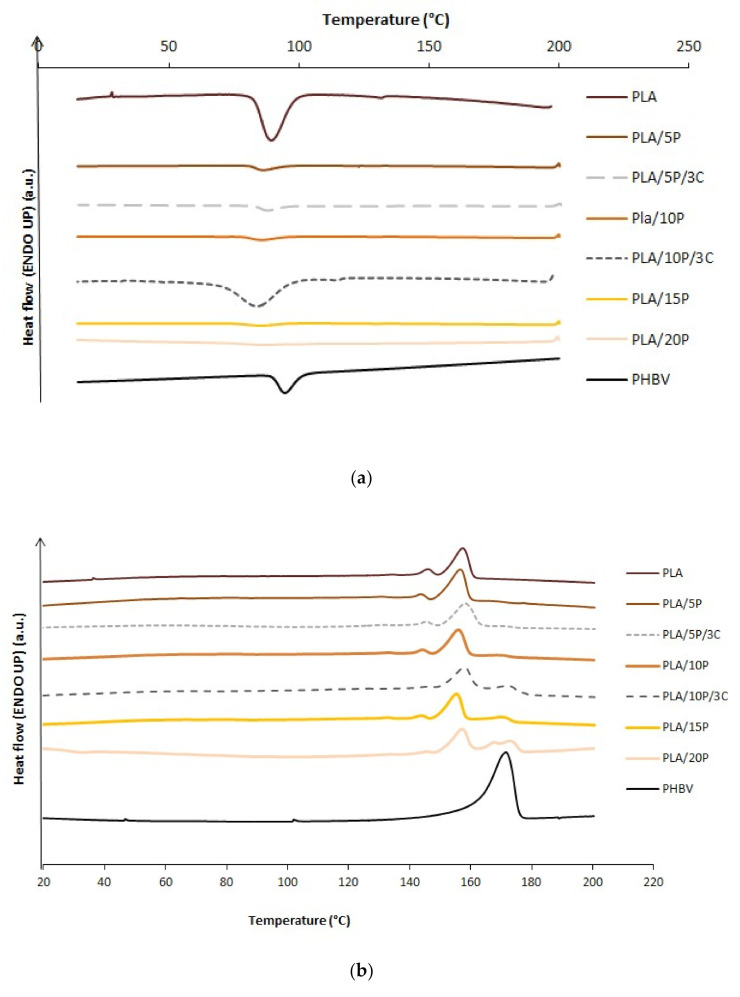
DSC curves of PLA, PHBV and their blends at (**a**) the cooling stage and (**b**) the second heating stage.

PLA and PHBV are typical semicrystalline polymers and their properties are highly related to their solid-state morphology and crystallinity. It is then important to study the influence of the minor phase on the crystallization of the matrix. Figure 3a shows the DSC cooling thermogram after being melted at 200 °C. A single crystallization peak was found at 94.7 (±0.3) °C and 89.4 (±0.5) °C for neat PHBV and PLA, respectively. The cold crystallization temperatures (T_c_) of PLA/PHBV blends were shifted to lower temperatures of 86.3 °C, 85.7 °C, 85.4 °C and 85.3 °C when the PHBV content increased from 5%, 10%, 15% and 20%, respectively. The presence of PLA restricted the crystallization of PHBV by suppressing the nucleation of PHBV in the blend. Similar results were also reported on the crystallization behavior of PHBV/PLA blends as the degree of crystallinity of PLA generally increased with PHBV [15,16]. For instance, the presence of 3 wt.% of C30B slightly affected the crystallization with an increase in the enthalpy from 15.5 J/g to 17.9 J/g for PLA/10/3C in comparison with PLA/10P. 

Figure 3b shows the DSC heating thermogram (second run) after cooling at 10 °C/min. In the case of PLA, the melting peak centered at 157.5 °C with a small shoulder at 145.9 °C, which corresponds to the melt of small and imperfect crystals of lower thermal stability [13]. PHBV presented also a single melting peak at 175.2 °C. Concerning the PLA/PHBV blends, a multi-step process was observed with up to three melting temperatures (T_m1_, T_m2_, and T_m3_) reported in Table 3, suggesting a lack of miscibility. The first double peak around 145–155 °C could be attributed to the formation of different crystal structures due to the melting of PLA, as reported in other PLA blends and composites [13,17]. The third peak could be the consequence of a recrystallization during melting attributed to possible PHBV degradation. As expected [8], the incorporation of C30B into the PHBV/PLA composite did not modify the melting temperatures of the blends. 

### 3.3. FTIR

**Figure 4:**
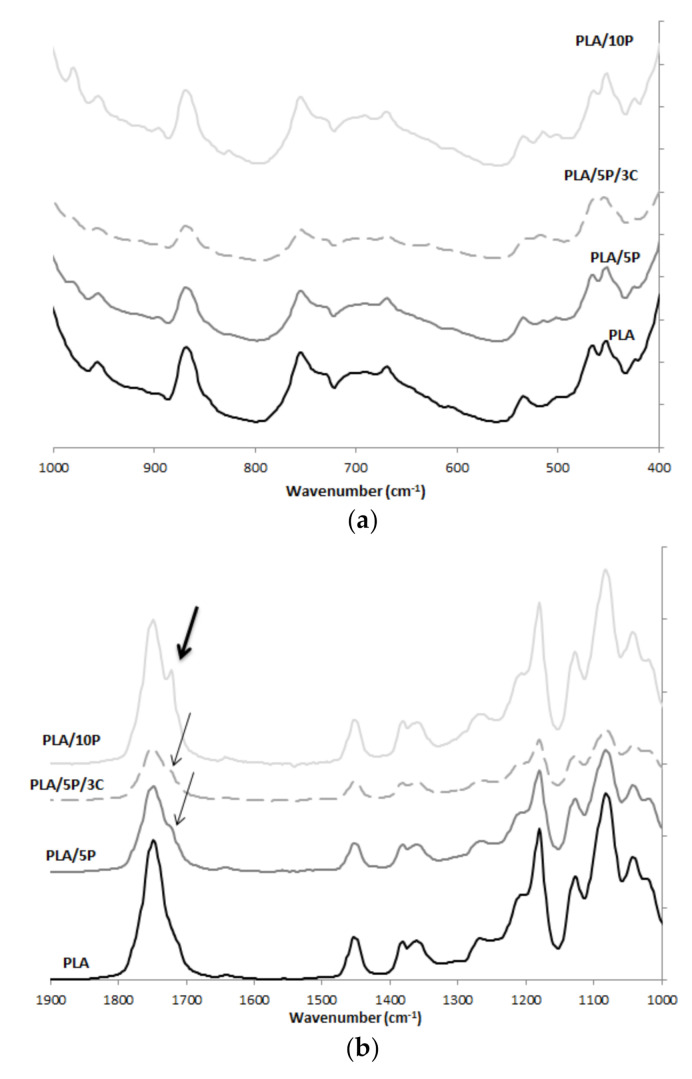
FT-IR spectra of PLA, PLA/5P, PLA/5P/3C and PLA/10P film samples in the range of (**a**) 1000–400 cm^−1^; (**b**) 1900–1000 cm^−1^.

FTIR spectra of some PLA/PHBV films are shown in Figure 4 with an arbitrary offset for comparison. All spectra displayed the characteristic bands of PLA-based materials. An intense peak was observed at 1749 cm^−1^, attributed to the carbonyl vibration in polyesters. The bands at 1180 cm^−1^ and 1083 cm^−1^ belong to asymmetric and symmetric C-O-C vibration, respectively. Two bands related to the amorphous and crystalline phases of PLA are found at 867 cm^−1^ and 755 cm^−1^. A peak located at 1724 cm^−1^ (Figure 4b, grey narrows) is increasingly intense, as the PHBV content increased as it belongs to stretching vibrations of the crystalline carbonyl group of PHBV [17], confirming the low miscibility of the two biopolyester. Regarding PLA/5P/3C, small intense peaks were observed at 519 cm^−1^ and 1035 cm^−1^, corresponding to Si-O bending and stretching, respectively, confirming the presence of C30B.

### 3.4. Contact Angle

**Figure 5:**
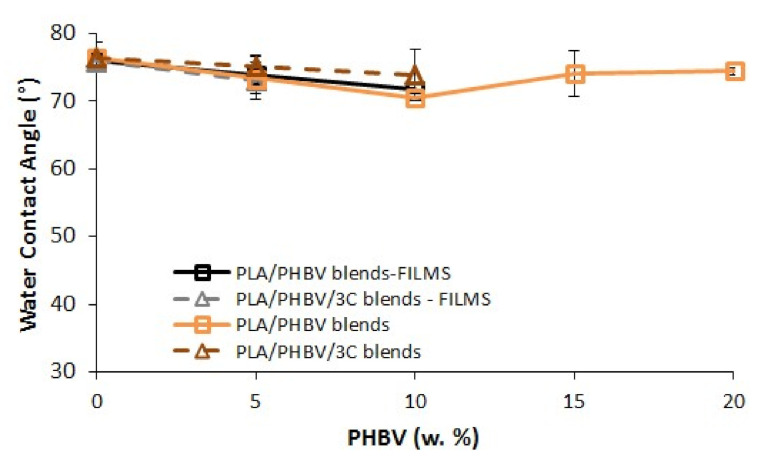
Water contact angle (WCA) of the bio-nanocomposites on films and dumbbell shape surfaces.

The water contact angle (WCA) measurements were investigated and results are shown in Figure 5. No significant differences of WCA were observed for neat PLA samples, either for the film or for the dumbbell shape. All values were in range of 76.1° ± 0.5. The wettability of the polymers is not changed by the addition of cloisite 30B. 

### 3.5. Tensile Properties

**Figure 6:**
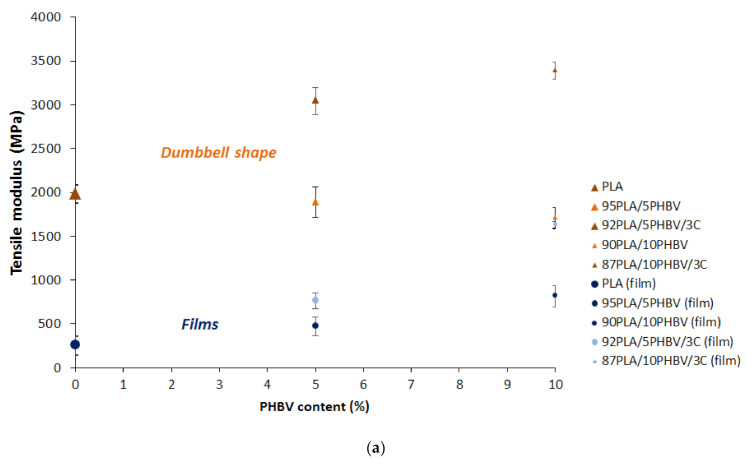

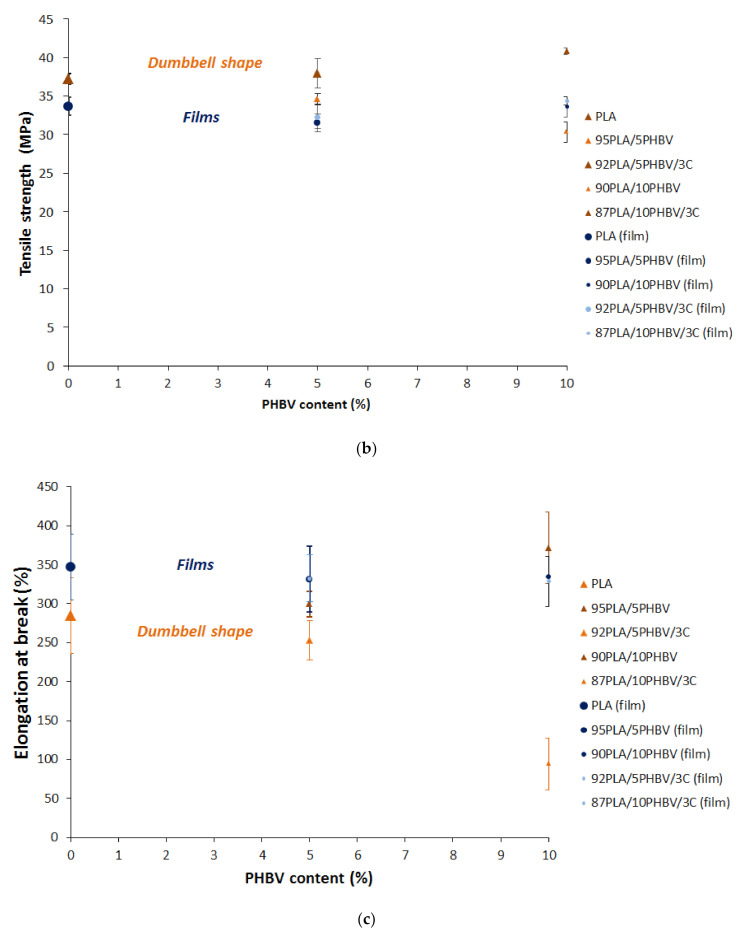
(**a**) Tensile modulus, (**b**) strength, and (**c**) elongation at break of neat PLA, PHBV, and their blends.

Figure 6 shows the tensile modulus, strength, and elongation at break of neat PLA, PHBV, and their blends (with and without C30B) according to PHBV content. As expected [17,18], the presence of plasticizer in this grade of PLA decreases both tensile strength and modulus compared to usual grades with elongation close to 5% [5]. Thereby, the high elongation at break (circa 350% for the films and 285% for dumbbell-shape samples) confirms the very good plasticizer efficiency, making it a good candidate when ductile behavior is required. However, it is obvious that the shape of the materials will highly influence their mechanical properties (Figure 6 and Table 4). Regarding dumbbell-shape PLA, the strength of the polymer is slightly higher (+11%) and its rigidity is clearly stronger (+686%) in comparison to the film, but the elongation at break is reduced (−18%). To a lesser extent, similar observations can be done for PLA/PHBV blends.

The addition of 5% and 10% of PHBV into the PLA matrix induced a diminution of the tensile properties of the blends (elongation and stress at break) whereas an increase in the Young modulus was observed. Similar results were reported regarding the diminution of the stress at break and the modulus. It is believed that the formation of spherulitic PHBV particles finely dispersed in the PLA matrix act as fillers [3,19].

Nevertheless, the addition of nanoclays helped to offset the loss of mechanical properties of the blends. The addition of 3% of C30B significantly increased the Young modulus of the blend measured at 3043 ± 157 MPa and 3386 ± 97 MPa for PLA/5PHBV and PLA/10PHBV, respectively, when the modulus of neat PLA was found to be 1981 ± 107 MPa for dumbbell-shape samples. The rigidity of the films also significantly increased with Young’s moduli of 252 ± 111 MPa, 762 ± 81 MPa, and 1630 ± 41 MPa for PLA, PLA/5P and PLA/10P, respectively. It might be due to a strong interfacial interaction between the polymers and the silicates layers leading to supramolecular assemblies. To a lesser extent, the maximal stress at break was slightly improved at the expense of a limited reduction in elongation with the addition of 3% of nanoclays. 

The formulation of the bio-nanocomposite PLA/10P/3C appears then as a promising candidate for sanitary products like wound dressing or face masks, which are required to fit the body shape. The mechanical properties of the films offered excellent rigidity (E = 1630 ± 41 MPa), good resistance (σ = 1630 ± 41 MPa), as well as considerable stretchability (ε = 329 ± 32%) in comparison with the limited ductility of the neat PLA (Table 4).

### 3.6. Electrospun Nanofibers

**Figure 7:**
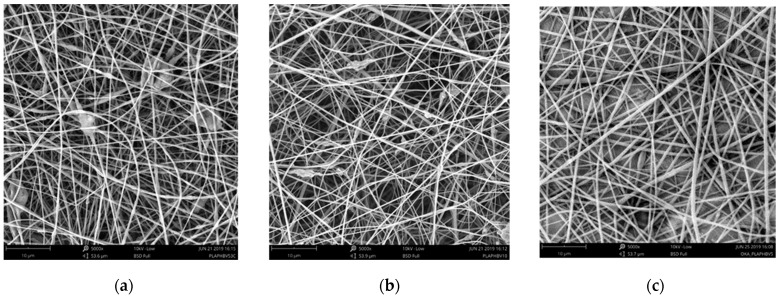
Electrospun nanofibers of (**a**) PLA/10P/3C; (**b**) PLA/10P and (**c**) PLA/5P observed by SEM.

The morphological aspects and the diameter of the electrospun fibers corresponding to the PLA/5P, PLA/10P blends and PLA/10P/3C nanocomposite compositions were investigated by SEM (Figure 7). PLA/PHBV blends have shown good spinnability with the formation of uniform, randomly oriented fibers, although some defects like beads are observed for the nanocomposite. The average fiber diameters were calculated as 324 ± 89 nm, 402 ± 113 nm and 433 ± 97 nm for PLA/10P/3C, PLA/10P and PLA/5P, respectively. The presence of the nanoclay provided narrower fibers. It is believed that the presence of modified montmorillonite with organic alkyl ammonium cations can improve the electrical conductivity, which may lead to smaller average fiber diameter. However, the SEM image in Figure 7a involving 3 wt.% of cloisite 30B indicates a non-homogeneous surface along the fiber, probably due to clay aggregation. 

## 4. Conclusions

Bionanocomposites based on plasticized PLA matrix, PHBV in a minor phase, and C30B as nanofiller were prepared. As expected, poor miscibility between PLA and PHBV was observed. The addition of PHBV up to 20 wt.% provoked a decrease in the thermal stability and has shifted crystallization and melting temperatures to lower values for all PLA/PHBV blends in comparison to the neat PLA matrix. Then, an increase in the material rigidity was observed with a significant reduction in the tensile strength. However, the large efficiency of the plasticized PLA matrix allowed elongation at break values close to 300% to be maintained. Meanwhile, the addition of 3 wt.% of clay C30B, well dispersed into the polymeric phase, did not significantly damage the thermal behavior of the blends but enabled a considerable increase in the mechanical properties of the blends. The formulation with moderate PHBV content (up to 10%) and reinforced with C30B appeared to be particularly interesting with balanced strength/elongation properties to provide stretchable and resistant films. Thus, the bionanocomposite could be a promising candidate to extend the functional properties of the biodegradable polymers, as well as for the production of electrospun nanofibers.

## Figures and Tables

**Table 1:** Bio-nanocomposites’ formulations.

Sample	PLA NF 141 (wt.%)	PHBV (wt.%)	C30B (wt.%)
PLA	100	-	-
PLA/5P	95	5	-
PLA/5P/3C	92	5	3
PLA/10P	90	10	-
PLA/10P/3C	87	10	3
PLA/15P	85	15	-
PLA/20P	80	20	-

**Table 2:** Decomposition temperatures at 5%, 10%, 50% weight loss and residues at 600 °C of the blends.

Sample	T_5%_ (°C)	T_10%_ (°C)	T_50%_ (°C)	Char_600 °C_ (%)
PLA	301	323	365	3.6
PLA/5P	302	314	364	3.4
PLA/5P/3C	300	317	365	5.8
PLA/10P	296	306	356	3.5
PLA/10P/3C	300	309	356	5.0
PLA/15P	291	303	358	2.9
PLA/20P	293	302	355	3.3
PHBV	287	291	306	1.3

**Table 3:** DSC properties of PLA, PHVB and their blends.

Sample	T_c_ (°C)	ΔH_c_ (J.g^−1^)	T_m1_ (°C)	ΔH_m1_ (J.g^−1^)	T_m2_ (°C)	ΔH_m2_ (J.g^−1^)	T_m3_ (°C)	ΔH_m3_ (J.g^−1^)
PLA	89.4	21.0	145.9	2.5	156.7	19.4	-	-
PLA/5P	86.3	20.3	143.7	1.6	156.3	22.6	-	-
PLA/5P/3C	87.4	18.2	145.3	1.5	158.2	18.4	171.8	0.50
PLA/10P	85.7	15.5	144.1	1.6	158.1	17.2	172.1	0.60
PLA/10P/3C	84.0	17.8	145.5	0.3	157.9	15.9	172.3	3.90
PLA/15P	85.4	15.9	144.0	1.1	155.4	16.7	171.6	3.40
PLA/20P	85.3	16.1	145.3	0.5	157.2	14.6	173.5	2.50
PHBV	94.7	76.9	138.1	9.8	172.5	80.4	-	-

**Table 4:** Effect of shape and nanoclay C30B on the tensile properties of PLA/PHBV blends.

Sample	Shape	C30B (%)	Tensile Strength (MPa)	Young modulus (MPa)	Elongation (%)
**PLA**	film	-	33.7 ± 0.7	252 ± 11	347 ± 49
	dumbbell	-	37.3 ± 1.2	1981 ± 107	285 ± 42
**95PLA/5PHBV**	film	-	31.5 ± 1.5	468 ± 66	331 ± 27
	film	3	32.3 ± 1.0	762 ± 81	333 ± 33
	dumbbell	-	34.7 ± 0.7	1888 ± 174	299 ± 16
	dumbbell	3	37.9 ± 1.9	3043 ± 157	253 ± 35
**90PLA/10PHBV**	film	-	33.6 ± 2.2	811 ± 87	334 ± 18
	film	3	34.4 ± 0.5	1630 ± 41	329 ± 32
	dumbbell	-	30.4 ± 1.3	1703 ± 123	371 ± 46
	dumbbell	3	40.8 ± 0.4	3386 ± 97	95 ± 33
